# 2. Predictors of Generalized Anxiety Disorder Symptoms in Residents of Fort McMurray Five Years after the Devastating Wildfires.

**DOI:** 10.1192/j.eurpsy.2023.268

**Published:** 2023-07-19

**Authors:** E. Owusu, R. Shalaby, E. Eboreime, N. Nkire, M. A. Lawal, B. Agyapong, G. Obuobi-Donkor, M. K. Adu, F. Oluwasani, W. Mao, H. Pazderka, V. I. Agyapong

**Affiliations:** 1 Department of Psychiatry, University of Alberta, Edmonton; 2 Department of Psychiatry, Dalhousie University, Halifax, NS, Canada

## Abstract

**Introduction:**

Natural disasters adversely impact individuals living in places where they occur, resulting in emotional distress. The wildfire that occurred in Fort McMurray (FMM), Alberta in 2016 is no different.

**Objectives:**

This study aims to identify the prevalence and predictors of Generalized Anxiety Disorder (GAD) symptoms in residents of FMM five years after the devastating wildfires.

**Methods:**

Data for the study were collected through a cross-sectional survey conducted online from the 24th of April to the 2nd of June 2021. A validated instrument, the GAD-7 scale, was used to collect information on anxiety.

**Results:**

Of the total number of 186 residents who took part in the study, the majority were females (85.5%), employed (94.1%), working at school boards (50.0%), and were either married, cohabiting, or partnered (71.0%). The prevalence of likely GAD among the study sample was 42.5%. Unemployed respondents were seventeen times more likely to develop GAD symptoms (OR = 16.62; 95% C.I. 1.23-223.67) while respondents who would like to receive mental health counseling were five times more likely to experience GAD symptoms (OR = 5.35; 95% C.I. 2.03-14.15). Respondents who suffered a loss of property because of the wildfire were two times more likely to develop GAD symptoms (OR = 2.36; 95% C.I. 1.01-22.62).

**Conclusions:**

Formulators of policy may mitigate GAD symptoms, particularly after natural disasters, by making long-term mental health counseling available and a key component of post-disaster management, and by investing in the social capital of the people to build resilience and support to deal with the post-disaster mental health effects.

**Disclosure of Interest:**

None Declared

